# Wheat Protein Hydrolysate Fortified With l‐Arginine Enhances Satiation Induced by the Capsaicinoid Nonivamide in Moderately Overweight Male Subjects

**DOI:** 10.1002/mnfr.201900133

**Published:** 2019-10-02

**Authors:** Verena Stoeger, Barbara Lieder, Johanna Riedel, Kerstin Schweiger, Julia Hoi, Veronika Ruzsanyi, Martin Klieber, Petra Rust, Joachim Hans, Jakob P Ley, Gerhard E Krammer, Veronika Somoza

**Affiliations:** ^1^ Department of Physiological Chemistry University of Vienna Althanstrasse 14 (UZA II) Vienna 1090 Austria; ^2^ Christian Doppler Laboratory for Bioactive Compounds Althanstrasse 14 (UZA II) Vienna 1090 Austria; ^3^ Institute for Breath Research University of Innsbruck Innrain 66 Innsbruck 6020 Austria; ^4^ Department of Nutritional Sciences University of Vienna Althanstrasse 14 (UZA II) Vienna 1090 Austria; ^5^ Symrise AG Research & Technology Flavors Division 37603 Holzminden Germany

**Keywords:** arginine, food satiety, gastric emptying, nonivamide, wheat protein hydrolysate

## Abstract

**Scope:**

Increasing the intake of satiety‐enhancing food compounds represents a promising strategy for maintaining a healthy body weight. Recently, satiating effects for the capsaicinoid nonivamide have been demonstrated. As various proteins and amino acids have also been demonstrated to decrease energy intake, oral glucose tolerance test (oGTT)‐based bolus interventions of 75 g glucose + 0.15 mg nonivamide (NV control) are tested with/without combination of a wheat protein hydrolysate (WPH: 2 g) and/or l‐arginine (ARG: 3.2 g) for their satiating effects in 27 moderately overweight male subjects.

**Methods and Results:**

Compared to NV control intervention, ARG and WPH + ARG treatment both reduce (*p* < 0.01) total calorie intake from a standardized breakfast by –5.9 ± 4.15% and –6.07 ± 4.38%, respectively. For the WPH + ARG intervention, increased mean plasma serotonin concentrations (AUC: 350 ± 218), quantitated by ELISA, and delayed gastric emptying, assessed by ^13^C‐Na‐acetate breath test (−2.10 ± 0.51%, *p* < 0.05), are demonstrated compared to NV control. Correlation analysis between plasma serotonin and gastric emptying reveals a significant association after WPH ± ARG intervention (*r* = –0.396, *p* = 0.045).

**Conclusion:**

Combination of WPH and ARG enhances the satiating effect of nonivamide, providing opportunities to optimize satiating food formulations by low amounts of the individual food constituents.

## Graphical abstract

A combination of satiating food constituents for enhancing satiety is a promising strategy for maintaining a healthy bodyweight. In this human intervention study, an l‐arginine‐enriched wheat protein hydrolysate is demonstrated to further increase the satiating effect of nonivamide, a pungent pepper constituent, in healthy male volunteers when administered as a protein shake prior to an ad libitum breakfast.

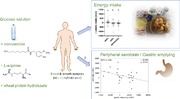

## 1 Introduction

Overweight, obesity, and associated diseases represent a rapidly growing problem in worlds’ population, causing burdens not only to the affected persons but also for the health care system.^1^ One feasible strategy that is currently in focus of research is to develop foods that combine nutrients to induce an early and more intensified feeling of satiety, leading to a reduced energy intake.^2^ The usage of an optimized combination of ingredients has the advantage that the satiating effect does not depend on the amount of a single compound, thereby limiting the risk of side or adverse effects. Such satiating products are thought to limit dissatisfaction during dieting, which often lead to poor compliance and effectiveness.

In general, feelings of satiety and hunger are regulated via a brain–gut signaling, which involves secretion of hormones and neurotransmitters from the stomach, duodenum, colon, and pancreas mediated by the hypothalamus.^3^, ^4^ Following food intake, blood glucose levels are regulated by the hormone insulin, which is immediately released from the pancreas to stimulate glucose uptake by peripheral tissues.^4^, ^5^ Moreover, intestinal satiety hormones such as Peptide YY (PYY), Glucagon‐like peptide‐1 (GLP‐1), and Cholecystokinin (CCK) are secreted in response to nutrient intake and delay gastric emptying, thereby leading to gastric distension and to satiation.^4^ Gastric emptying is also regulated by peripheral serotonin, a neurotransmitter stored and released by enterochromaffin cells of the intestine.^6^ Central serotonin, in addition, also promotes satiety.^7^, ^9^

Food intake is a complex process that is regulated by psychological and physiological factors. Whereas short‐term regulation of food intake involves mechanisms that are effective during a meal to promote satiation and thereby terminate food intake, long‐term factors require continuous metabolic activities that mediate satiety and determine the time between meals. From a physiological perspective, the feeling of hunger represents the need for food and is controlled by central and peripheral signals. Central factors include activation of hypothalamic neuropeptide Y (NPY)/agouti‐related protein (AgRP) neurons and GLP‐1 neurons in the nucleus of the solitary tract that project directly to the ventral tegmental area and nucleus accumbens to control food intake. Moreover, gastric afferent nerve fibers and stretch receptors translate the state of full‐ or emptiness of the stomach to the hypothalamus region in the brain. In contrast, peripheral factors include the secretion of gastrointestinal and peripheral hormones, as well as nutrient concentrations in the blood. High concentrations of amino acids, fatty acids, and glucose suppress the feeling of hunger, whereas a secretion of the hormone ghrelin is known to stimulate food intake. Furthermore, ghrelin stimulates gastric emptying,^10^ which is suggested to be mediated via activation of the vagus nerve or ghrelin receptors. In addition, ghrelin has been shown to be involved in the inhibition of insulin release via central melanocortin signaling.^11^

Consumption of proteins has been associated with prolonged feelings of satiety in comparison to fats and carbohydrates when applied in equicaloric amounts.^12^ Moreover, protein consumption is known to retard gastric emptying^13^, ^14^, ^15^, ^16^ and to stimulate gastric acid secretion (GAS).^15^ Secretion of gastric acid facilitates the degradation of proteins into amino acids, which may contribute to the satiating effect of proteins. Dietary l‐arginine, i.e., has been shown to reduce energy intake in rodents^16^ and to delay and inhibit gastric emptying.^17^ Furthermore, we recently demonstrated that l‐arginine stimulated mechanisms of GAS^18^ and increased the release of serotonin in a parietal cell model.

Besides proteins, also pungent compounds, e.g., capsaicin and its less pungent structural analogue nonivamide, have been well investigated for their satiating effects. Studies from our own group demonstrated a reduced energy intake and accompanied by increased plasma serotonin levels after a 0.15 mg bolus administration of nonivamide in a cross‐over human intervention trial.^19^ Furthermore, administration of a milk shake fortified with 0.15 mg nonivamide for 12 weeks to overweight, but otherwise healthy subjects, increased peripheral serotonin levels and prevented a dietary‐induced body fat gain.^20^ Also, on the cellular level, nonivamide has been shown to promote serotonin secretion by neural SH‐SY5Y cells.^21^

Since it has been demonstrated that a combination of satiety enhancing ingredients can be a feasible strategy for combating overweight and obesity,^2^ in the current study, a combination of a wheat protein hydrolysate and l‐arginine was tested on its potential to enhance the satiating effect of nonivamide. The amino acid l‐arginine was chosen due to its published benefits on obesity by decreasing plasma glucose levels and fatty acids,^22^ but also by its ability to reduce food intake.^16^ Moreover, since a satiating potential of proteins is well known, we investigated whether a fortification of a wheat protein hydrolysate with l‐arginine additionally enhances satiety. A wheat protein hydrolysate was chosen since wheat protein presents the lowest amount of l‐arginine among dietary legumes and grains,^23^, ^24^ allowing to study the satiating effect of l‐arginine fortification. Moreover, wheat is one of the world's most commonly consumed cereal grains. The amount of wheat protein hydrolysate administered as a bolus was limited to 2 g due to its unpleasant taste, although other proteins for which a satiating effect has been shown in previous studies had been administered in higher amounts.^25^ For l‐arginine, an amount of 3.2 g l‐arginine was chosen, which is also in accordance with common doses used for dietary l‐arginine supplementation,^26^ and is far below the No Observed Adverse Effect Level (NOAEL) of 30 g d^–1^ for healthy young adults.^26^

The aim of the present study was to investigate whether a combination of a wheat protein hydrolysate and l‐arginine enhances the satiating effect of nonivamide in a short‐term human intervention study with healthy male subjects. Hence, in this study, we hypothesized a wheat protein hydrolysate fortified with l‐arginine enhances the satiating effect of nonivamide when administered in an oral glucose tolerance test (oGTT).

## 2 Experimental Section

### 2.1 Characteristics of the Subjects

From December 2017 to April 2018, a total of 50 men were recruited for a medical screening by advertisements in web forums and billboards at Universities in Vienna. An age between 20 and 45 years, a BMI between 25 and 30 kg m^–^², but neither tobacco consumption nor alcohol abuse or medical treatment were allowed for metabolically healthy, sensorially untrained study subjects. Subjects with a BMI > 25 kg m^–^² were categorized as moderately overweight, although body composition (fat mass, free fatty mass) was not considered. Due to estrogen fluctuations during the menstrual cycle and their influence on serotonin levels, women were excluded from this study.^27^, ^28^ Within the medical screening after recruitment, hematological parameters, plasma thyroid hormone status, plasma lipids, and glucose concentrations after an oGTT were analyzed by Ihr Labor 1220 (Medical diagnostics laboratory including microbiology, Dr. Gabriele Greiner, Vienna, Austria) to ensure recruited subjects were metabolically healthy. Moreover, a fasting blood glucose < 120 mg dL^–1^ was mandatory for enrolment. Body height was measured by a stadiometer (Seca, Germany) with precision of 0.1% and body weight was assessed by a digital scale within 100 g exactly (Weinberger, Germany). By means of the open source software GPower 3.1, a number of 50 volunteers was identified when a statistically significant difference of total energy intake of 0.49 MJ between two dependent means and a power of 80% was taken into account. The effect size of 0.49 MJ was based on one of our previous human intervention studies, in which the satiating effect of a 0.15 mg nonivamide bolus administration was investigated and demonstrated to reduce total energy intake from an ad libitum breakfast from 4.75 ± 0.45 MJ after control intervention to 4.26 ± 0.33 MJ.^23^ Hence, 50 male volunteers were recruited and a total of 27 volunteers completed the study per protocol (**Table** 1).

**Table 1:** Study subjects‘ characteristics

*N*	27
Gender	male
Age [years]	25.7 ± 0.7
Height [m]	1.82 ± 0.0
Body weight [kg]	89.0 ± 2.1
BMI [kg m^–^²]	26.8 ± 0.4
fasting blood glucose [mg dL^–1^]	74.4 ± 1.4

Data are depicted as mean ± SEM.

Overall, the study protocol adhered to the guidelines of the Declaration of Helsinki. Although dietary intervention studies at the University of Vienna do not require approval by the local ethics board, the study protocol using the pungent compound nonivamide was approved by the ethics committee of the city of Vienna (registration no. EK 12–084–0812).

### 2.2 Dosage Information/Dosage Regimen’ in the Experimental Section

In the current human intervention study, a bolus of 0.15 mg nonivamide^19^ alone or in combination with either 2 g of a wheat protein hydrolysate and/or 3.2 g l‐arginine was administered to healthy young males in an oGTT (75 g glucose/300 mL water). Participants were asked to drink the test solution within 5 min.

The bolus administration of 0.15 mg nonivamide had been chosen as reference intervention since it had previously been demonstrated to effectively decrease energy intake and increase plasma serotonin concentrations in healthy volunteers, whereas post‐load plasma concentrations were below the limit of detection (5 fMol).^19^ Although data on habitual dietary intake of nonivamide are lacking, a daily intake of total capsaicinoids between 0.5 and 4 mg kg^–1^ body weight has been estimated by the European Council.^29^

Nonivamide (Flavor & Extract Manufacturers Association (FEMA) 2787) is classified as a Generally Recognized As Safe (GRAS) aroma compound by the Food and Drug Administration (FDA), and can be used for food fortification up to 10 ppm.^30^ According to risk assessment published by the European Food Safety Agency (EFSA), the maximum daily intake is 6 µg per capita.^34^ Furthermore, a NOAEL (No Observed Adverse Effect Level) of 8.4 mg kg^–1^ body weight d^–1^ has been established.^31^ In the current study, the amount of 0.15 mg nonivamide applied together with a 300 mL glucose solution correspond to 0.5 mg L^–1^ or 0.5 ppm or 500 ppb. Since this concentration is below the NOAEL and also meets the FDA recommendations for food fortification, the nonivamide in the amount administered did not bear any risk to the study subjects. Because of its limited solubility in water, the administered amount of 150 µg nonivamide was pre‐dissolved in 15 µL ethanol absolute. The final concentration of the ethanol in the study solution was <0.001% and can also not be considered as harmful to the study participants.

The amount of 2 g of the wheat protein hydrolysate (food‐grade quality) applied in this study was assessed in sensorial pretests and found to be palatable (data not shown). According to regulation no. 1169/2011 by the EFSA, wheat protein hydrolysate is not subjected to food declarations, although its content as food allergen has to be labeled.^32^

In addition to nonivamide and a wheat protein hydrolysate 3.2 g of free l‐arginine HCl (FEMA 3819) were administered. The amount of 3.2 g l‐arginine was chosen, since this is a common amount for l‐arginine supplements and is in the range of habitual daily intake of up to 5 g.^33^

### 2.3 Study Design

**Figure 1:**
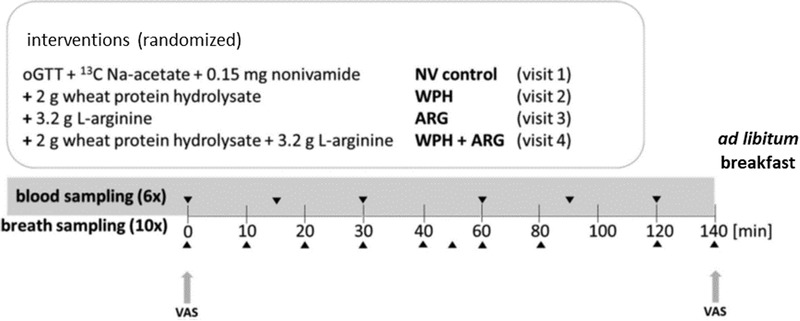
Study design of this cross‐over human intervention study. A total of 27 volunteers underwent each of the four interventions with a 7 day interval between interventions and set their subjective feeling of hunger by means of a visuale analgoue scale (VAS).

A single blinded cross over study with four randomized groups according to four interventions (**Figure** 1) was conducted. Test subjects received the following interventions in a randomized order on four study days after an overnight fast, applied within an oGTT: Nonivamide control (**NV**, 0.15 mg nonivamide, 75 g glucose, 15 µL ethanol, and 150 mg ^13^C‐Na‐acetate), wheat protein hydrolysate (**WPH**) (2 g wheat protein hydrolysate 0.15 mg nonivamide, 75 g glucose, 15 µL ethanol, and 150 mg ^13^C‐Na‐acetate), l‐arginine hydrochloride (**ARG**) (3.2 g l‐arginine, 0.15 mg nonivamide, 75 g glucose, 15 µL ethanol, and 150 mg ^13^C‐Na‐acetate) or **WPH +ARG** (2 g wheat protein hydrolysate, 3.2 g l‐arginine, 0.15 mg nonivamide, 75 g glucose, 15 µL ethanol, and 150 mg ^13^C‐Na‐acetate). The four visits were carried out 1 week apart. In addition, the test subjects were asked not to consume foods containing naturally higher amounts of ^13^C, e.g., corn, tequila, or cane sugar, 48 h prior to intervention. On each study day, subjects were asked to rate their subjective feeling of hunger on a 100 mm visual analogue scale (VAS), followed by collection of two baseline breath samples and baseline blood collection. Further blood samples were collected 15/30/60/90 and 120 min after administration of the oGTT. In parallel, breath samples were collected every 10 min during the first hour, followed by time points 80/120 and 140 min. After the last breath sample was taken, the subjective feeling of hunger was assessed again by means of a VAS, and a standardized continental breakfast with ≈3000 kcal was served ad libitum. The breakfast consisted of: four rolls, three slices whole grain rye bread, 3.5 slices of cheese (≈125 g), eight slices of ham (≈99 g), 80 g butter, 100 g jam, 60 g honey, three pieces of coffee cream, 4 g sugar, 180 g yoghurt, 200 mL water, and 200 mL coffee or tea. Short‐term energy intake from the ad libitum breakfast was analyzed using nut.s v1.32.50 by weighing the remaining food on each subject's tray. Dietary intake data were collected by performing repeated 24‐h‐recalls using the software GloboDiet. GloboDiet was developed by the International Agency for Research on Cancer (IARC) within the European Investigation into Cancer and Nutrition Study (EPIC‐Study) and adopted to Austrian nutrition habits by the Department of Nutritional Sciences to carry out standardized 24‐h‐recalls. The reported foods assessed during the interviews were linked to the German food composition database Bundeslebensmittelschlüssel 3.02.^34^, ^35^

### 2.4 Blood Sample Collection

Venous blood samples for plasma serotonin concentrations were collected in EDTA‐coated tubes and immediately centrifuged at 1800 × *g* at 4 °C for 15 min. For avoiding platelets in plasma, samples for serotonin were additionally centrifuged for 1 min at 7000 x *g* at 4 °C. For increasing ghrelin stability during storage, whole blood samples were mixed with AEBSF (4‐[2‐aminoethyl benzene] sulfonyl fluoride, Sigma, Germany), centrifuged at 1800 × *g* at 4 °C for 15 min and stabilized with hydrochloric acid (Sigma, Germany).

### 2.5 Plasma Concentrations of Total Ghrelin, Total GLP‐1, Serotonin, Insulin, and Glucose

Ghrelin (LOD: 50 pg mL^–1^) and GLP‐1 (LOD: 2 pm) were determined by means of a sandwich ELISA (Merck Millipore, Germany), whereas serotonin was quantified by means of a competitive ELISA (LOD: 2.6 ng mL^–1^). Serotonin samples under this LOD were additionally analyzed using the serotonin high sensitive ELISA with an LOD of 0.39 pg per sample (both DLD Diagnostika, Hamburg, Germany). Insulin concentrations were assessed using a sandwich ELISA (LOD: 50 pg mL^–1^) obtained from IASON (Graz, Austria). Glucose concentrations were quantitated by a colorimetric assay, presenting an LOD of 0.23 mg dL^–1^ (Cayman Europe, Tallinn, Estonia).

### 2.6 Gastric Emptying

The study drink was labeled with ^13^C‐Na‐acetate,^36^ produced according to the guidelines for clinical trial material by Euroisotop (France). Breath bags for sample collection were obtained from Cambridge Isotope Laboratories, Inc. (Massachusetts, USA). Breath sample collection was standardized as follows: after the breath was hold for 20 s, the breath bag had to be fully inflated by one subsequent exhale. Twenty‐six subjects per intervention provided adequate breath samples (CO_2_ > 3 vol%) in which ^12^CO_2_ and ^13^CO_2_ concentrations were quantitated by infrared spectroscopy (POCone, Otsuka Pharmaceutical S.A., Barcelona, Spain).

In detail, each study subject had to fill two baseline breath bags (breath air before intervention was applied) and sample breath bags (breath air containing ^13^CO_2_) for every time point (*t*_10_–*t*_140_). These bags were analyzed and the device detected DOB (Delta Over Baseline)‐values ([^13^CO_2_/^12^CO_2_]_sample _– [^13^CO_2_/^12^CO_2_]_baseline sample_; *n* = 22–26 in Figure S6 and *n* = 1 in Figure S5, Supporting Information) and the ^12^CO_2_‐concentrations (%) of the sample as well as the baseline. For considering the body surface area of each study subject, the DuBois formula^37^ was used and the ^13^CO_2_ in mol m^–2^/precursor in mol (%) of the DOB‐value was calculated, based on these data the area under the curve (AUC) as well as the ΔAUC (subtracting the NV control from each intervention) were generated and are plotted as **Table** 2 and Figure S6, Supporting Information.

**Table 2:** Influence of WPH and ARG alone or in combination on Δ DOB AUC considering the body surface of each study subject, n = 22–26. Data are presented as ΔAUC values of the Delta Over Baseline (DOB) presenting ^13^CO_2_/^12^CO_2_ ratio and are depicted as mean ± SEM

Intervention	∆DOB AUC [treatment – NV control]	*p*‐Value [*t*‐test vs NV control]
WPH	–1000 ± 343	0.01
ARG	–634 ± 398	0.12
WPH+ARG	–1214 ± 295	0.001

### 2.7 Statistical Analyses

Statistical analyses were performed by SigmaPlot 12 and figures were generated either by SigmaPlot 12 or GraphPad Prism 8. A normal distribution of the data was determined by Shapiro–Wilk test. Statistical differences were defined with a *p*‐value < 0.05. To test if WPH + ARG increased the satiating effect in comparison to NV, a one‐tailed *t*‐test or Mann–Whitney rank sum test, for non‐normally distributed data sets, was conducted. Intergroup differences of WPH, ARG and WPH + ARG and NV were tested by means of a One Way ANOVA or a One Way ANOVA on Ranks followed by Student–Newman–Keuls method post hoc, respectively. Δ‐Values were calculated by subtracting the NV control from the treatment. For serotonin and gastric emptying, total AUC was calculated over time. A correlation between serotonin and gastric emptying was assessed by Pearson Product Moment Correlation. Calculation of individual ^13^CO_2_ was performed by using DuBois formula.^37^

## 3 Results

### 3.1 Assessing the Subjective Feeling of Hunger by Means of VAS

**Figure 2:**
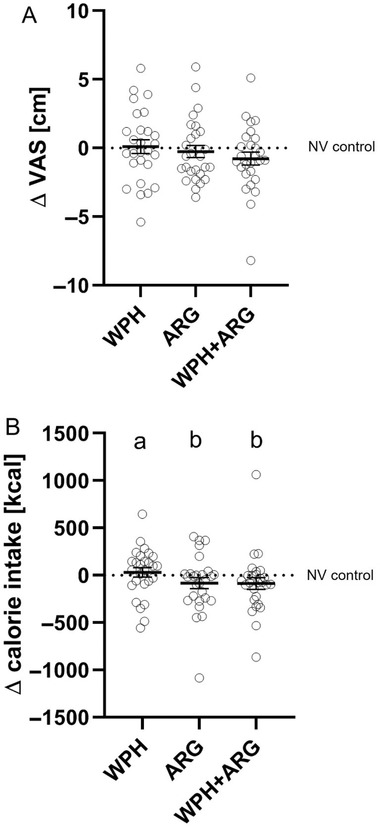
A) Differences in subjective ratings of hunger before and after administration of 75 g glucose solution supplemented with either 0.15 mg NV (control), and 2 g wheat protein hydrolysate (WPH) and/or 3.2 g l‐arginine (ARG), were adjusted to values for NV control ( = 0; *n*  =  27). Group mean of WPH+ARG was significantly lower than those of NV control as identified by Mann–Whitney Rank Sum Test (*p* < 0.05). Every symbol represents a study participant and means are shown as a black line. B) Total calorie intake from a standarized ad libitum breakfast served 140 min after oral adminstration of 75 g glucose in combination with either NV (NV control), WPH, ARG, or WPH + ARG in 27 healthy males. Letters indicate significant differences between treatments by One Way ANOVA on Ranks followed by Student–Newman–Keuls post hoc test, *p* < 0.05. Group mean of WPH+ARG (–86 ± 63 kcal) was decreased by –6.07 ± 4.38 % compared to NV control as identified by Mann–Whitney Rank Sum test (*p* < 0.01). Total kcal intake of the NV control group ranged between 753 to 2067 kcal. Every symbol represents a study participant and means are shown as a black line.

In comparison to the NV control (mm after intervention – mm before intervention =  Δmm set to 0 for calculating the effects of WPH, ARG, and WPH+ARG interventions), ARG and the combination of WPH + ARG decreased the subjective feeling of hunger (ARG Δ: −0.97 ± 0.31, WPH + ARG Δ: −0.57 ± 0.31, p < 0.05) (**Figure** 2A).

### 3.2 Total Energy Intake After an Ad Libitum Breakfast

ARG as well as WPH + ARG intervention decreased total energy intake from the standardized breakfast in comparison to NV control treatment, with mean reductions of –83 ± 60 kcal (−5.9 ± 4.15%, *p* < 0.05) and 86 ± 63 kcal (−6.07 ± 4.38%, *p* < 0.05). Moreover, the WPH + ARG intervention decreased energy intake more effectively than the WPH intervention (*p* < 0.05; Figure 2B). Total kcal intake from the standardized ad libitum breakfast in the NV control group ranged between 753 to 2067 kcal.

### 3.3 Plasma Concentrations of Glucose, Insulin, Ghrelin, and GLP‐1

**Figure 3:**
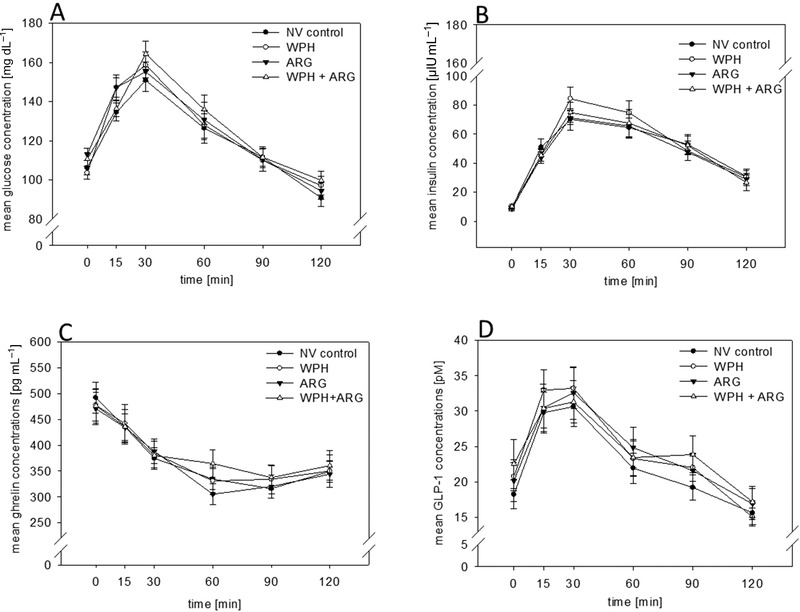
A–D) Administration of WPH, ARG and its combination WPH + ARG did not influence plasma glucose A), insulin B), ghrelin C), and GLP‐1 D) concentration (*p* > 0.05). Statistical significances were tested by One Way ANOVA and *t*‐test on plasma concentrations over time as well as on AUC values.

Statistical analysis demonstrated no intergroup differences for plasma glucose, insulin, ghrelin, and GLP‐1 levels after WPH, ARG, and WPH + ARG intervention compared to NV control (*p* > 0.05; **Figure** 3A–D).

### 3.4 Plasma Serotonin Levels

**Figure 4:**
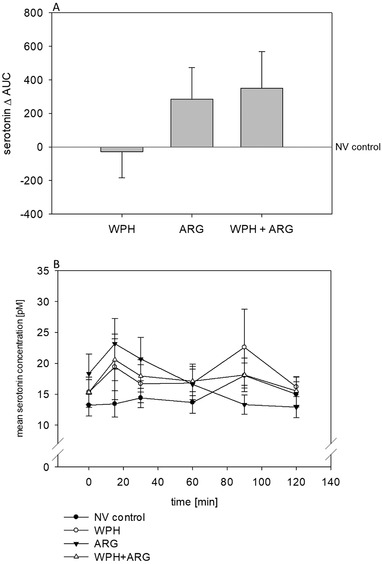
A) ARG + WPH intervention increased plasma serotonin levels (expressed as AUC [pm × min]) in 27 healthy volunteers over time in comparison to NV control (WPH + ARG: 350 ± 218, NV set to 0). Statistical significance between treatments was calculated by One Way ANOVA (*p* > 0.05). Group mean after WPH+ARG intervention was significantly higher than that after NV control treatment as identified by Mann–Whitney Rank Sum test (*p* < 0.05). Values are shown as mean ± SEM. Plasma serotonin concentrations of the NV control group ranged from 0.40 to 42 pg mL^–1^. B) Influence of NV control, WPH, ARG, and WPH + ARG intervention on plasma serotonin levels. Statistical significances were tested by One Way ANOVA and *t*‐test on plasma concentrations over time as well as on AUC values (*p* > 0.05). Plasma serotonin concentrations are depicted as mean ± SEM.

WPH + ARG intervention increased Δ‐plasma AUC values up to 350 ± 218 in comparison to NV control intervention set to 0 (*p* < 0.05; **Figure** 4A). No intergroup differences were calculated by means of ANOVA (Figure 4B). Serotonin concentrations in subjects of the NV control group ranged from 0.40 to 42 pg mL^–1^.

### 3.5 Gastric Emptying

By using DuBois^37^ formula considering body surface and Nalimov outlier test, a delay in gastric emptying was detected in 22–26 subjects after WPH as well as WPH + ARG intervention, in comparison to NV control intervention (Δ AUC: WPH: 1000 ± 343 ≙ –1.72 ±  0.60%, *p* < 0.01; WPH + ARG: 1213 ± 295 ≙ –2.10 ±  0.51%, *p* < 0.001; Table 2). ANOVA analyses did not reveal any intergroup differences.

### 3.6 Pearson Correlation Between Gastric Emptying and Plasma Serotonin

**Figure 5:**
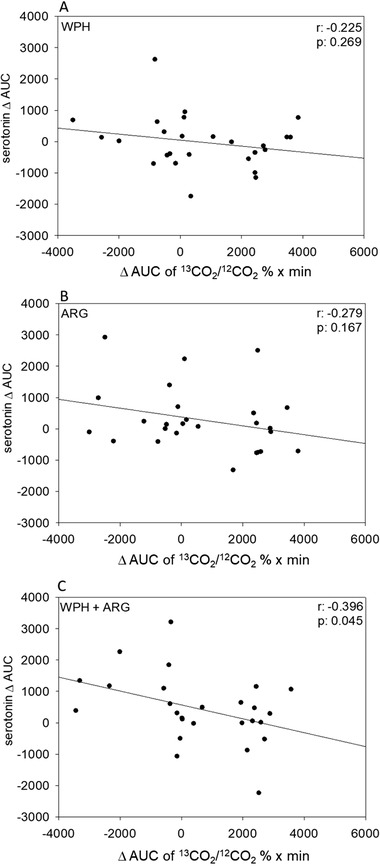
A–C) Correlation analysis between Δ AUC plasma serotonin and ∆ AUC of ^13^CO_2_/^12^CO_2_ % × min rates (NV control was subtracted from interventions) after WPH, ARG, or WPH + ARG intervention. Correlation coefficients were computed by means of Pearson Product‐Moment Correlation method. For intervention, a significant correlation was demonstrated, with WPH+ARG (*r* = −0.396, *p* = 0.045).

Correlation analysis by means of Pearson Product Moment Correlation between ∆AUC of ^13^CO_2_/^12^CO_2_ % × min  and serotonin revealed a significant association for the WPH + ARG intervention (*r* = –0.396, *p* = 0.045; **Figure** 5A–C).

### 3.7 Twenty‐Four Hours Calorie Intake Calculated by Globodiet

Only WPH + ARG intervention reduced the calorie intake 24 h post load in comparison to NV control intervention (Δ_before – after_ kcal intake: –360 ± 147; *p* < 0.05) (**Table** 3).^34^, ^35^ No intergroup differences were calculated by means of ANOVA. The total mean calorie intake of the subjects after NV control intervention was 2951 ± 141 kcal.

**Table 3:** A decrease of the total calorie intake on the study day was detected by the 24‐h‐recall method GloboDiet, Mann–Whitney Rank Sum Test showed a significance of *p* < 0.05, *n* = 27. Data are presented as mean ± SEM. Δ Kcal has been calculated by subtracting NV control from interventions. The NV control intervention resulted in a mean kcal intake of 2951 ± 141 kcal

Intervention	Δ Intake [Kcal]	*p*‐Value [*t*‐test vs NV control intervention]
WPH	–249 ± 149	0.459
ARG	–487 ± 170	0.082
WPH+ARG	–360 ± 147	0.025

## 4 Discussion

Dietary intake of foods containing constituents to promote satiation present a promising strategy to reduce daily energy intake and to combat the increasing problem of obesity.^2^, ^38^ The aim of the current study was to identify whether the addition of a wheat protein hydrolysate and l‐arginine, or a combination thereof, enhances the satiating effect of the pungent compound NV. This structural analogue of capsaicin has recently been shown to decrease the subjective feeling of hunger, to reduce total energy intake from a standardized breakfast, and to increase plasma serotonin levels when applied in an amount of 0.15 mg in an oGTT.^19^ In this study, total energy intake and satiety‐associated gastro‐intestinal and peripheral signals were investigated after administration of 0.15 mg NV (control intervention) in combination with 2 g wheat protein hydrolysate (WPH intervention) and/or 3.2 g l‐arginine (WPH + ARG or ARG intervention) concomitant with 75 g glucose as a caloric load. The amount of WPH administered as bolus dose was limited to 2 g due to its unpleasant taste, although other proteins for which a satiating effect has been shown in previous studies, were administered in higher amounts.^25^ A wheat protein hydrolysate was chosen due to its low l‐arginine content in comparison to other proteins,^23^, ^24^ and was therefore suitable for investigating the satiating effect of an l‐arginine fortification. Currently, a NOAEL of dietary added l‐arginine up to 30 g d^–1^ is proposed for healthy young adults.^26^ For l‐arginine, an amount of 3.2 g was chosen, which is also in accordance with common amounts used for dietary l‐arginine supplementation.^26^ Outcome measures included the subjective feelings of hunger, the total energy intake right after an ad libitum breakfast as well as over a period of 24 h post‐intervention. In addition, changes in plasma glucose, insulin, serotonin, GLP‐1, and ghrelin concentrations were analyzed over time for 140 min after intervention. Furthermore, all interventions included the administration of ^13^C‐Na‐acetate for analyzing the ratio of ^13^CO_2_/^12^CO_2_ in breath samples as an indicator of gastric emptying.

For ARG and WPH + ARG interventions, a reduced subjective feeling of hunger in comparison to the NV control intervention was demonstrated, indicating a satiating effect of which the effect size was similar for both interventions. Whether bolus amounts of >2 g of a wheat protein hydrolysate with or without l‐arginine fortification could further decrease the subjective feelings of hunger needs to be investigated in future studies.

The demonstrated effect of the ARG and WPH + ARG intervention is reflected by a lower energy intake calculated from the remaining food after a standardized ad libitum breakfast 2 h post load compared to NV control intervention. Plasma concentrations of glucose, insulin, ghrelin, and GLP‐1 concentrations did not change after any of the intervention, which has been previously demonstrated for nonivamide in a comparable study design.^19^ Since only 27 study subjects completed all four interventions, whereas G‐Power analysis had revealed a number of 50 study participants, the low samples size of the study might have limited the effects on plasma hormones. Furthermore, the low number of study participants might be one reason for the reduced effect size of the ad libitum energy intake from the standardized breakfast of 0.17 MJ, in comparison to the expected effect size of 0.47 MJ. Overall, these results indicate a satiating effect of wheat protein hydrolysate and l‐arginine, which is in accordance with current literature, stating that protein intake^39^ in human and l‐arginine^16^ in rodents, induces a decrease in energy intake.

In addition to a reduction of the subjective feeling of hunger and energy intake, plasma serotonin levels in this study were also increased after WPH + ARG intervention. We previously demonstrated that administration of 0.15 mg nonivamide increased plasma serotonin levels after a bolus dose,^19^ as well as after a daily dose in a 12 week human intervention study.^20^ In the present study, we demonstrate plasma serotonin levels to further increase after administration of a combination of 0.15 mg nonivamide with either 3.2 g l‐arginine and/or 2 g wheat protein hydrolysate. These results indicate a role of l‐arginine not only on energy intake, but also on plasma serotonin levels. A sustained increase of central serotonin release after l‐arginine superfusion has been demonstrated by Sinner and colleagues.^40^ In addition, there is also growing evidence for peripheral serotonin to promote satiety via nerval activation, although its satiating pathways are not yet fully understood.^7^, ^41^ Moreover, there is growing evidence supporting a regulating role of peripheral serotonin in the systemic energy homeostasis.^7^, ^41^ In one of the previous studies, an oral application of 50 mg encapsulated l‐arginine resulted increased plasma serotonin levels in healthy subjects.^42^ Also the data of the present study strengthen the hypothesis of a modulating role of peripheral serotonin on energy homeostasis. Up to now, it can just be speculated how l‐arginine and a wheat protein hydrolysate stimulate the release of serotonin from enterochromaffin (EC) cells. In the gastrointestinal tract, EC cells act also as nutrient sensors. Hence, it could be hypothesized that EC cells act as peptide and amino acid sensors, which might respond by 5‐hydroxytryptamine (5‐HT) release during meal intake. A significant release of 5‐HT after a protein‐rich meal in comparison to carbohydrate‐ or fat‐rich meals was demonstrated by Blum and colleagues.^43^ Recently, Lund and colleagues suggested a paracrine mechanism for stimulating EC cells by nutrient metabolites in the gut.^44^ Furthermore, an increased 5‐HT release could be mediated by an increased Ca^2+^ influx via voltage activated calcium channels,^45^ which might have been induced by WPH+ARG in this study. Extracellular serotonin concentrations are regulated by serotonin‐selective reuptake transporters (SERT) to cross the plasma membrane, where it is enzymatically degraded.^46^ From the current human intervention study, it can be hypothesized that ARG and WPH may partly inhibit der SERT system, possibly resulting in increased plasma serotonin concentrations, which are currently discussed to promote satiety in addition to central serotonin.^41^

Peripheral serotonin is also involved in the control of gastric motility,^47^ which regulates gastric empyting.^48^ A delay in gastric emptying is known to promote early satiety.^49^ However, evidence for l‐arginine's effect on gastric emptying in humans is still conflicting, by describing a delay^17^, ^50^ on the one hand, and an accelerated gastric emptying^51^ on the other, which may be due to application of different detection methods and study models. In addition, proteins differentially regulate gastric emptying depending on their origin, with a mostly increased gastric emptying for whey protein in comparison to cod, gluten, or casein, when administrated within a standardized test meal in an amount of 45 g.^14^ In the present human intervention study, both the WPH as well as the WPH + ARG intervention delayed gastric emptying, which was analyzed using ^13^C‐Na‐acetate as a marker.

The satiating effect of a wheat protein with and without l‐arginine fortification demonstrated in our study is suggested to be supported by plasma serotonin levels correlating with gastric emptying. Furthermore, our results indicate that high plasma serotonin levels are favorable for a delay in gastric emptying. Thus, we hypothesize that the increased plasma serotonin levels lead to a retard in gastric emptying, which promotes the feeling of satiety and helps to reduce total energy intake. However, further research investigating the interaction between gastric emptying and plasma serotonin levels is needed. Overall, the combined administration of nonivamide, l‐arginine, and wheat protein hydrolysate in an oGTT was most effective in triggering this proposed satiety cascade, demonstrating the importance of an optimized nutritional composition of foods to achieve the most effective satiating effect.

To summarize, in the present human intervention study, we demonstrate that the combined bolus administration of 0.15 mg nonivamide, 3.2 g l‐arginine and 2 g wheat protein hydrolysate in an oGTT reduced subjective feelings of hunger and total energy intake from a standardized breakfast, which might be due to increased plasma serotonin levels that lead to a delay in gastric emptying. Combinations of nutrients are favorable for potentiating the satiating effects of foods without having to apply high concentrations of the individual ingredients in the food's formulation. The use of nonivamide, for example, in large amounts, is limited due to its pungency,^19^ and excessively high‐protein diets may pose long‐term health risks to the kidneys,^52^ whereas a common side effect of high arginine doses above 9 g d^–1^ is diarrhea.^53^ In the present study, each active agent was applied in an amount that is unlikely to induce any adverse effects, and the combined application of nonivamide, arginine, and wheat protein hydrolysate can be hypothesized to be more effective than the individual compounds.

Future studies including women are needed for a more representative study population to substantiate the demonstrated effect as basis for the development of satiating food formulations. In addition, long‐term effects on satiety signals and body composition have not been investigated yet, but are needed to conclusively assess the efficacy of this approach to maintain a healthy body weight.

## Conflict of Interest

The authors J. Hans, J. P. Ley, G. E. Krammer are employees at Symrise AG, Holzminden, Germany, which holds IP on nonivamide in the context of satiety management.

## Supporting information

Supporting Information
