# Supplementary material for: Wheat Protein Hydrolysate Fortified With l‐Arginine Enhances Satiation Induced by the Capsaicinoid Nonivamide in Moderately Overweight Male Subjects
Source: Mol Nutr Food Res. 2019 Oct 2;63(23):1900133. doi: 10.1002/mnfr.201900133 (PMC6916637; doi:10.1002/mnfr.201900133)
Supplement: Supplementary file 1 — Supporting Information [file MNFR-63-na-s001.doc]

**Supplemental Material to**

**Wheat protein hydrolysate fortified with L-arginine enhances satiation induced by the capsaicinoid nonivamide in moderately overweight male subjects**

Verena Stoeger^2^, Barbara Lieder^1^, Johanna Riedel^1^, Kerstin Schweiger^1^, Julia Hoi^2^, Veronika Ruzsanyi^3^, Martin Klieber^3^, Petra Rust^4^, Joachim Hans^5^, Jakob P Ley^5^, Gerhard E Krammer^5^, Veronika Somoza^1,2†^

^1^Department of Physiological Chemistry, University of Vienna, Althanstrasse 14 (UZA II), Vienna 1090, Austria; ^2^Christian Doppler Laboratory for Bioactive Compounds, Althanstrasse 14 (UZA II), Vienna 1090, Austria; ^3^Institute for Breath Research, University of Innsbruck, Innrain 66, Innsbruck 6020, Austria; ^4^Department of Nutritional Sciences, University of Vienna, Althanstrasse 14 (UZA II), Vienna 1090, Austria; ^5^Symrise AG, Research & Technology Flavors Division, P.O. Box 1253, 37603 Holzminden, Germany

**Figure S1**: Influence of NV control, WPH, ARG and WPH + ARG on subjective feeling of hunger, which was assessed before and 140 min after the intervention. After NV control and WPH intervention, there was a stronger increase in the subjective feeling of hunger. Statistical differences were tested by Mann-Whitey Rank Sum Test (p < 0.05) and are marked by *. One Way ANOVA indicated no intergroup differences.

**Figure S2**: There was no influence induced by NV control, WPH, ARG and WPH + ARG interventions on total kcal intake from an *ad libitum* standardized breakfast, when statistical performances were done with absolute calorie values. Mann-Whitey Rank Sum Test and One-Way ANOVA indicated no differences. (p > 0.05) Mean values are depicted as dashed line.


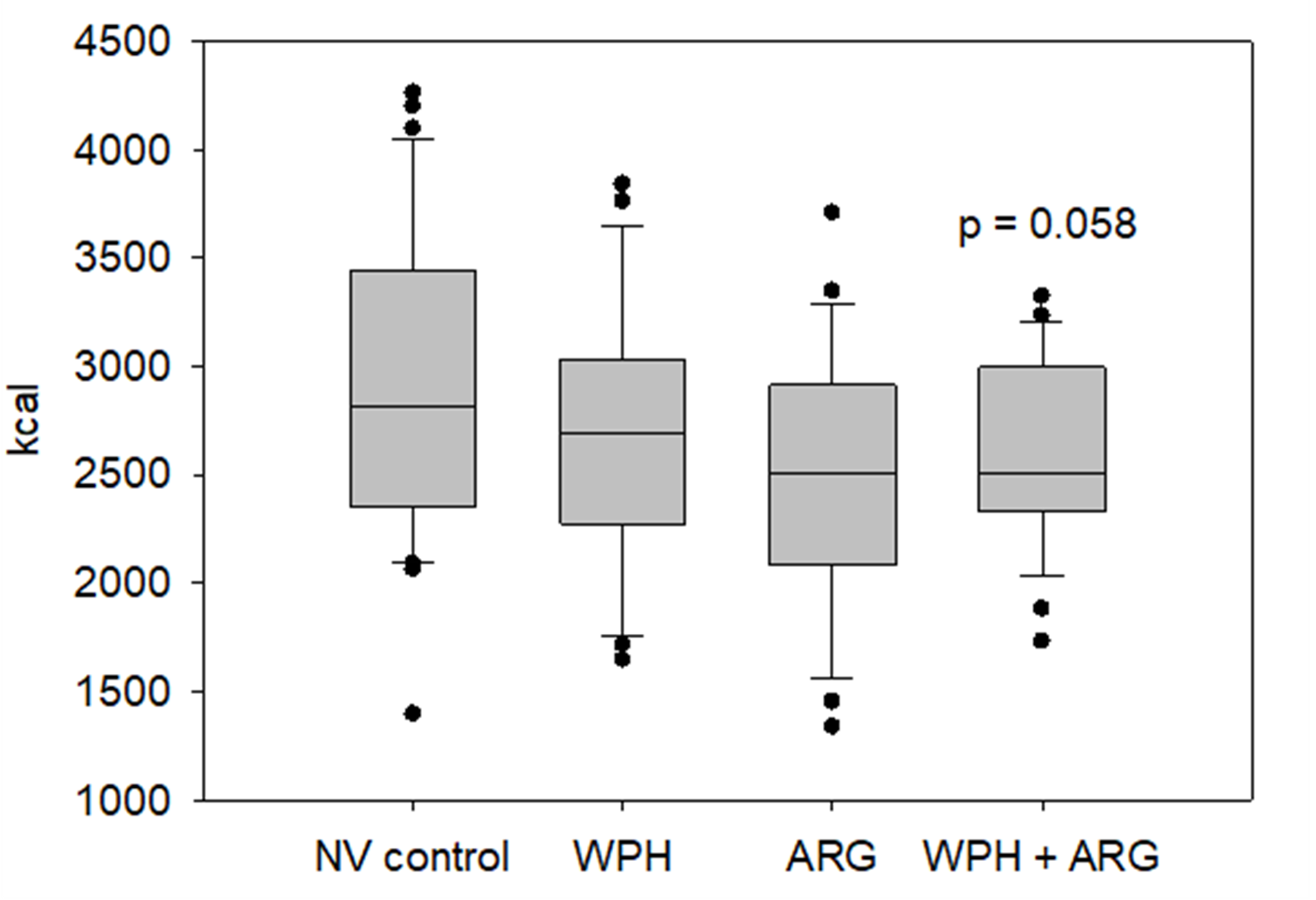


**Figure S3**: Total calorie intake on the study day assessed by means of the 24h recall method GloboDiet. For WPH + ARG treatment, a p-for trend (p = 0.058) was detected. Mann-Whitey Rank Sum Test and One Way ANOVA indicated no differences. (p > 0.05)

**Figure S4**: Delta Over Baseline (DOB) values in % over time after administration of the NV control, WPH, ARG and the combination WPH + ARG. n = 27. One Way ANOVA analyses did not reveal any significances. DOB % values are depicted as mean ± SEM.

**Figure S5**: Delta Over Baseline (DOB) values in % over time of one participant after administration of NV control, WPH, ARG and the combination WPH + ARG. n = 1


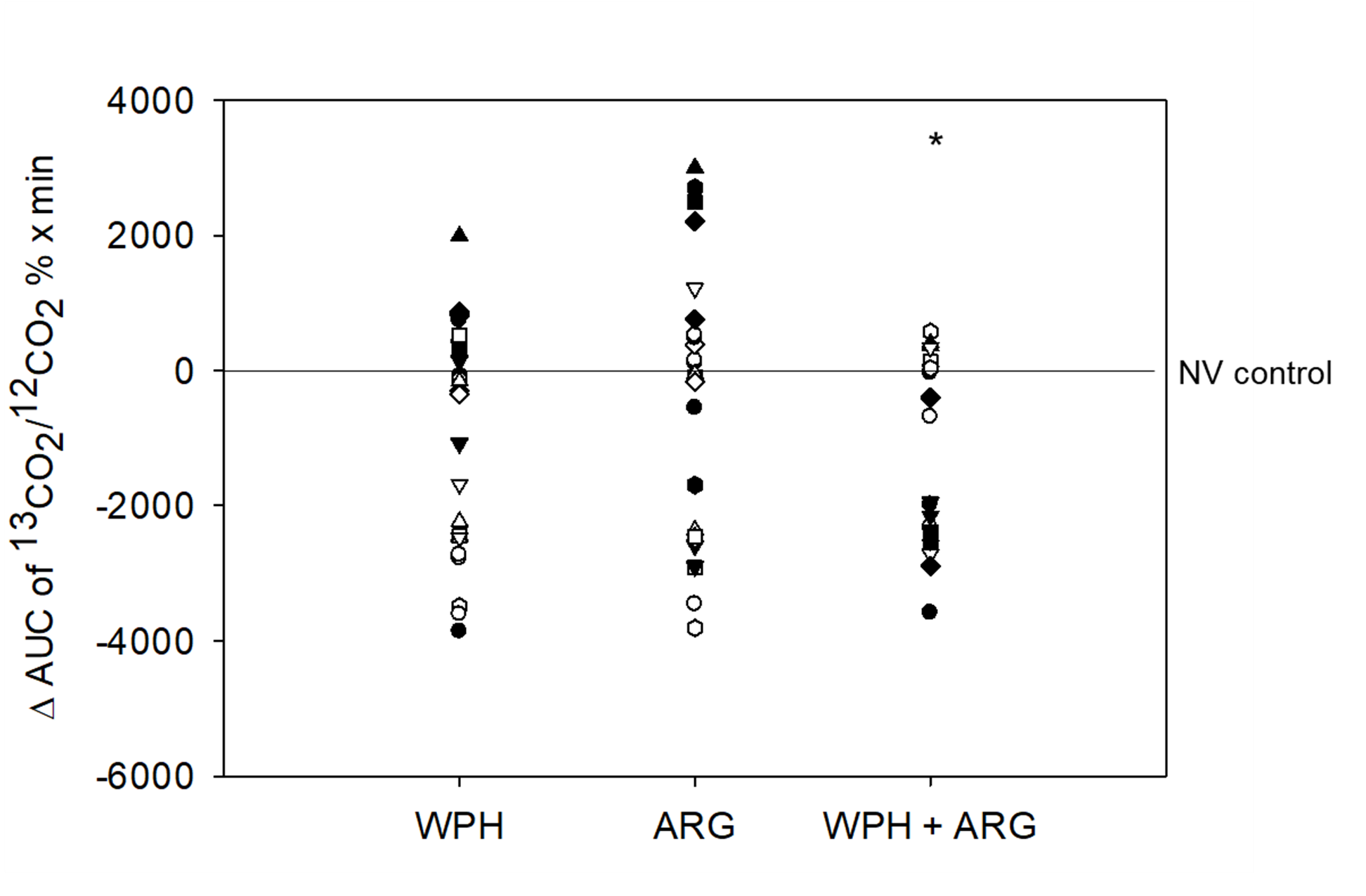


**Figure S6**: WPH + ARG treatment delayed gastric emptying in comparison to the NV control in healthy volunteers. Asterisk (*) indicate significance tested by Mann-Withney Rank Sum Test (p <0.05). No intergroup differences were detected after conduction of a One-Way ANOVA. Δ DOB AUC values are depicted as mean ± SEM.
